# Multi-domain effectiveness of guselkumab evaluated via composite indices through 1 year in patients with PsA and inadequate response to TNFi: *post hoc* analysis of COSMOS

**DOI:** 10.1093/rheumatology/keae586

**Published:** 2024-10-22

**Authors:** Laure Gossec, Xenofon Baraliakos, Daniel Aletaha, Mohamed Sharaf, Emmanouil Rampakakis, Frédéric Lavie, Clementina López-Medina, Carlo Selmi, Laura C Coates

**Affiliations:** INSERM, Institut Pierre Louis d’Epidémiologie et de Santé Publique, Sorbonne Universite, Paris, France; APHP, Rheumatology Department, Pitie Salpetriere Hospital, Paris, France; Rheumazentrum Ruhrgebiet, Ruhr University Bochum, Herne, Germany; Division of Rheumatology, Department of Medicine III, Medical University of Vienna, Vienna, Austria; Johnson & Johnson, Middle East FZ LLC, Dubai, UAE; Department of Pediatrics, McGill University, Montreal, QC, Canada; JSS Medical Research Inc., Scientific Affairs, Montreal, QC, Canada; The Janssen Pharmaceutical Companies of Johnson & Johnson, Paris, France; Reina Sofía University Hospital, IMIBIC, University of Córdoba, Córdoba, Spain; Rheumatology and Clinical Immunology, Humanitas Research Hospital, Rozzano, Milan, Italy; Rheumatology and Clinical Immunology, Humanitas University, Milan, Italy; Nuffield Department of Orthopaedics, Rheumatology and Musculoskeletal Sciences, University of Oxford, Oxford, United Kingdom

**Keywords:** Guselkumab, psoriasis, composite indices, low disease activity, remission, tumour necrosis factor inhibitor, COSMOS

## Abstract

**Objective:**

Evaluate guselkumab efficacy, an anti-interleukin-23p19-subunit antibody, in patients with active psoriatic arthritis (PsA) and inadequate response to 1 or 2 tumour necrosis factor inhibitors (TNFi-IR), utilizing composite indices assessing disease activity across disease domains.

**Methods:**

In the Phase IIIb COSMOS trial, 285 adults with TNFi-IR PsA were randomized (2:1) to receive guselkumab 100 mg or placebo at Week (W)0, W4, then every 8 weeks through W44. Patients receiving placebo crossed over to guselkumab at W24. In this *post hoc* analysis, composite indices evaluated included the Disease Activity Index for Psoriatic Arthritis (DAPSA), Disease Activity Score 28 (DAS28), Psoriatic Arthritis Response Criteria (PsARC), Psoriatic Arthritis Disease Activity Score (PASDAS), GRAPPA Composite score (GRACE), modified Composite Psoriatic Disease Activity Index (mCPDAI), minimal disease activity (MDA), and very low disease activity (VLDA). Through W24, treatment failure rules were applied. Through W48, non-responder imputation was used for missing data.

**Results:**

Greater proportions of guselkumab- than placebo-randomized patients achieved composite index endpoints relating to low disease activity (LDA; 14.8–52.4% *vs* 3.1–28.1%) or remission (3.7–5.3% *vs* 0.0–2.1%) at W24. Among guselkumab-randomized patients, LDA rates increased to W48 (DAPSA, 44.4%; DAS28, 47.8%; PASDAS, 34.4%; GRACE, 33.3%; mCPDAI, 40.2%), and 27.0% and 64.0% achieved MDA and a PsARC response, respectively. In the placebo→guselkumab crossover group, W48 response rates were similar to the guselkumab-randomized group.

**Conclusion:**

Guselkumab treatment provided substantial benefits across multiple disease domains, with increasing proportions of patients achieving LDA/remission over 1 year, highlighting the effectiveness of guselkumab despite previous inadequate response to TNFi.

Rheumatology key messages

- Composite indices evaluate disease activity across multiple PsA disease domains, and include both patient-reported and physician-assessed outcomes.
- Guselkumab treatment resulted in sustained low disease activity/remission of PsA over 1 year, as assessed via an extensive set of composite indices.
- Guselkumab effectively treats the diverse manifestations of PsA, including in patients with inadequate response to TNF inhibitors.

## Introduction

Psoriatic arthritis (PsA) is a chronic inflammatory disease characterized by several distinct clinical manifestations, including peripheral arthritis, axial disease, dactylitis, enthesitis, skin disease, and nail disease [1, 2].

Among patients with PsA whose disease can no longer be controlled by conventional synthetic disease-modifying antirheumatic drugs (csDMARDs), current treatment guidelines recommend the use of a biologic disease-modifying antirheumatic drug (bDMARD) [1, 2]. As such, patients with PsA are often treated with tumour necrosis factor (TNF) inhibitors (TNFi) [3]. However, a large proportion of patients receiving their first TNFi do not achieve a ≥20% improvement in American College of Rheumatology criteria (ACR20 response) within 6 months of treatment [3]. Further, among patients with an inadequate response (IR) or intolerance to their first TNFi, those who switch to a second TNFi may have significantly poorer responses than those who do not switch [4–6]. As such, treatment guidelines generally support only one switch within the TNFi class before treatment with a drug with an alternative mechanism of action [2].

Owing to the heterogeneous and systemic nature of PsA, treatment recommendations now include a *treat-to-target* approach, with the goal of achieving remission or low disease activity (LDA) across all domains of the disease [1, 2, 7, 8]. Composite indices provide a method to comprehensively assess disease activity using patient-reported and physician-assessed outcomes across multiple domains of PsA, and they are applicable to both clinical trials and real-world clinical practice [9–11]. Some of these composite indices have been adapted from other diseases, such as rheumatoid arthritis (e.g. the Disease Activity Score 28-joint count [DAS28]). Other composite indices, like the Disease Activity in PsA (DAPSA), were developed for use in PsA but focus primarily on the joints, while others created specifically for PsA evaluate a greater number of disease domains, reflective of the heterogeneous nature of the disease. These include the Psoriatic Arthritis Response Criteria (PsARC), Psoriatic Arthritis Disease Activity Score (PASDAS), Group for Research and Assessment of Psoriasis and Psoriatic Arthritis (GRAPPA) Composite score (GRACE), and the Composite Psoriatic Disease Activity Index (CPDAI) [12, 13].

Guselkumab is a human monoclonal antibody that targets the p19 subunit of interleukin (IL)-23, and has indications in both PsA and psoriasis [14, 15]. In the Phase IIIb randomized, placebo-controlled COSMOS clinical trial in adults with active PsA, the efficacy and safety of guselkumab was evaluated in patients with an IR (inadequate efficacy or intolerance) to one or two TNFi (TNFi-IR PsA) [16]. The primary end point of ACR20 response at Week 24 was met, and response rates and mean improvements for the signs and symptoms of PsA were maintained or improved through 1 year in the guselkumab group [16].

Owing to the multi-domain nature of PsA and the large proportion of patients in clinical practice who are receiving TNFi or who have TNFi-IR PsA, there is an unmet need to evaluate the efficacy of bDMARDs across disease domains in this patient population [16, 17]. In this *post hoc* analysis of COSMOS, we assess the efficacy of guselkumab across multiple PsA disease domains through 48 weeks, utilizing various clinically relevant composite indices and composite scores, in patients with TNFi-IR PsA.

## Methods

### Patients

Eligible adults were diagnosed with PsA and fulfilled the ClASsification criteria for Psoriatic ARthritis (CASPAR) at screening. Patients had active disease (tender joint count [TJC] and swollen joint count [SJC] both ≥3) and active (≥1 psoriatic plaque of ≥2 cm) or documented history of plaque psoriasis or current nail psoriasis, and had demonstrated a lack of benefit or intolerance to one or two TNFi. Patients could continue stable baseline use of methotrexate, sulfasalazine, hydroxychloroquine, leflunomide, oral corticosteroids, and non-steroidal anti-inflammatory drugs/other analgesics. Targeted synthetic disease-modifying antirheumatic drugs (tsDMARDs) were prohibited before and during study participation. Patients with active tuberculosis were excluded, and patients with latent tuberculosis received appropriate prophylaxis.

### Trial design

COSMOS was a Phase IIIb, randomized, double-blind, placebo-controlled clinical trial (NCT03796858) conducted at 84 European sites from March 2019 to November 2020; the overall COSMOS trial design has been reported previously [16]. Briefly, adults were randomized 2:1 to receive guselkumab 100 mg at Weeks 0 and 4, then every 8 weeks through Week 44 (final assessment at Week 48), or to receive placebo at Weeks 0, 4, 12 and 20 with crossover to receive guselkumab 100 mg at Weeks 24, 28, 36 and 44. Patients with <5% improvement from baseline in both TJC and SJC at Week 16 qualified for early escape; those receiving guselkumab continued randomized treatment (including a placebo dose at Week 16 to maintain blinding), while those in the placebo group received guselkumab at Weeks 16, 20, then every 8 weeks through Week 44. Early escape patients could initiate or increase the dose of one permitted concomitant medication up to the maximum allowed dose at the physician’s discretion.

COSMOS was conducted in accordance with the Declaration of Helsinki and Good Clinical Practice guidelines, and all participants provided written informed consent. The trial protocol was approved by the governing ethical body at each site.

### Assessments

Efficacy assessments and outcome measures in the COSMOS trial have been previously reported; DAPSA LDA, DAPSA remission, minimal disease activity (MDA), and very low disease activity (VLDA) composite indices were presented at Weeks 24 and 48 [16]. In this *post hoc* analysis, we report further composite indices (and their individual components), including DAS28 LDA, PsARC, PASDAS LDA and VLDA, GRACE LDA and modified CPDAI (mCPDAI) LDA (Table 1).

**Table 1.** Components of composite indices

Composite index	Components or outcomes	Score calculation and disease state threshold	Timepoints assessed in COSMOS (Weeks)
DAPSA	TJC (0–68) SJC (0–66) Patient pain assessment (0–100 mm VAS) PtGA of arthritis activity (0–100 mm VAS) CRP (mg/dL)	Higher DAPSA score represents more severe disease activity. LDA and remission defined as scores of ≤14 and ≤4, respectively [13]	4, 8, 12, 16, 20, 24, 48
DAS28	TJC (0–28) SJC (0–28) CRP (mg/dL) PtGA of disease activity (0–100 mm VAS)	Higher DAS28 score represents more severe disease activity. LDA defined as a score of <3.2 [18, 19]	4, 8, 12, 16, 20, 24, 48
PsARC	TJC (0–68) SJC (0–66) Patient’s overall assessment of disease activity (arthritis, VAS) Physician’s overall assessment of disease (VAS)	Improvement in ≥2 of the following criteria, including ≥1 of the joint criteria, with no deterioration in the other criteria: [20] ≥30% decrease in SJC ≥30% decrease in TJC ≥20% improvement in the patient’s overall assessment of disease activity ≥20% improvement in the physician’s overall assessment of disease	4, 8, 12, 16, 20, 24, 48
PASDAS	Physician global assessment of skin and joints (0–100 mm VAS) PtGA of skin and joints (0–100 mm VAS) SF-36 PCS (0–100) SJC (0–66) TJC (0–68) LEI (0–6) Tender dactylitis count (0–20) CRP (mg/L)	Total score ranges from 0 to 10, with higher scores representing more severe disease activity. PASDAS LDA and VLDA defined as scores of ≤3.2 and ≤1.9, respectively [13, 17]	8, 16, 24, 48
GRACE	TJC (0–68) SJC (0–66) HAQ-DI (0–3) PtGA of disease activity (arthritis and psoriasis, 0–100 mm VAS) Patient assessment of skin disease activity (0–100 mm VAS) PtGA of disease activity (arthritis, 0–100 mm VAS) PASI (0–72) PsAQOL score	The AMDF is calculated by transforming the component scores using predefined algorithms and expressing the score as a mean with a score of 0–1, where 1 indicates a better state than 0. Total GRACE score = (1−AMDF)×10. GRACE LDA was defined as a score of ≤2.3 [13]	16, 24, 48
mCPDAI	TJC (0–68) SJC (0–66) HAQ-DI (0–3) PASI (0–72) Dactylitis Enthesitis	Within each domain, a score (0–3) was assigned according to predefined cutoffs and summed to yield a total score (0–12), where a higher score represents more severe disease activity. mCPDAI LDA defined as a score of ≤3.2 [13]	16, 24, 48
	mCPDAI excluded the axial disease domain, and thus assessed four domains (joints, skin, entheses and dactylitis)		
MDA and VLDA	TJC ≤1 SJC ≤1 LEI ≤1^a^ PASI ≤1 Patient pain VAS ≤15 mm PtGA (arthritis and psoriasis) VAS ≤20 mm HAQ-DI ≤0.5 [13, 17]	Patients fulfilled MDA or VLDA criteria if they achieved five (MDA) or seven (VLDA) of the seven listed outcomes [13, 17]	16, 24, 48

a In COSMOS, enthesitis was assessed (as part of MDA/VLDA) using the LEI. The LEI has demonstrated significant agreement with other measures of enthesitis (e.g. MASES), and is considered easier to implement in clinical practice than other enthesitis measures [21].

Assessment weeks for the multi-domain composite indices were determined based on the common measurement schedule for the individual components.

AMDF: arithmetic mean of the desirability function; CRP: C-reactive protein; DAPSA: Disease Activity Index for Psoriatic Arthritis; DAS28: Disease Activity Score 28; GRACE: Group for Research and Assessment of Psoriasis and Psoriatic Arthritis (GRAPPA) Composite score; HAQ-DI: Health Assessment Questionnaire Disability Index; LDA: low disease activity; LEI: Leeds Enthesitis Index; MASES: Maastricht Ankylosing Spondylitis Enthesitis Score; mCPDAI: modified Composite Psoriatic Disease Activity Index; MDA: minimal disease activity; PASDAS: Psoriatic Arthritis Disease Activity Score; PASI: Psoriasis Area and Severity Index; PsAQOL: Psoriatic Arthritis Quality of Life Index; PsARC: Psoriatic Arthritis Response Criteria; PtGA: patient global assessment; SF-36 PCS: 36-item Short Form Health Survey physical component summary score; SJC: swollen joint count; TJC: tender joint count; VAS: visual analogue scale; VLDA: very low disease activity.

### Statistical analyses

In this *post hoc* analysis, we evaluated the proportions of patients achieving various endpoints over time. The time points assessed were determined by the first common assessment time of a composite index’s components. Efficacy analyses were conducted using the full analysis set from the COSMOS clinical trial, which included all randomized patients who received ≥1 dose of the study agent. Patients with missing data and those who met treatment failure criteria through Week 24 (i.e. discontinued study agent and/or study participation for any reason, initiated or increased the dose of allowed csDMARDs or oral corticosteroids for PsA, initiated protocol prohibited medications/therapies for PsA, or met early escape criteria [16]) were considered non-responders (i.e. non-responder imputation [NRI]/composite NRI).

Treatment group comparisons at each time point through Week 24 were conducted using logistic regression, in which achievement of each end point (including the components of each composite index) at each visit through Week 24 was the dependent variable. Baseline use of csDMARDs (yes *vs* no), number of prior TNFi (one *vs* two), baseline score of the respective index, and treatment assigned (guselkumab *vs* placebo) were used as covariates. *P*-values through Week 24 were not adjusted for the multiplicity of testing.

For patients randomized to placebo, only those who crossed over to guselkumab at Week 24 as planned were included in the Week 48 analyses. The proportion of patients achieving each end point (and the associated components of each composite index) after Week 24 through Week 48 is described using NRI only for missing data (i.e. with no treatment failure rules) for both the guselkumab-randomized and the placebo→guselkumab groups. To assess the maintenance of response, the proportions of guselkumab-randomized patients achieving each composite response at both Week 24 and Week 48 were determined.

## Results

### Baseline demographics and disease characteristics

At Week 0, 285 patients with TNFi-IR PsA were randomized to guselkumab (*n *=* *189) or placebo (*n *=* *96) [16]. At Week 16, 39 (21%) patients in the guselkumab arm and 45 (47%) patients in the placebo arm qualified for early escape. Through Week 24, 15 (8%) and 8 (8%) patients in the guselkumab and placebo groups, respectively, discontinued treatment. Overall, 51 (53%) placebo-randomized patients switched to guselkumab at Week 24 as planned. In total, 167 (88%) patients randomized to guselkumab and 83 (86%) placebo→guselkumab patients completed the study treatment to Week 44 [16].

Baseline demographics and disease characteristics were generally similar between the placebo and guselkumab groups (Table 2) [16]. On average, patients had PsA for ∼8 years, with active skin disease (mean Psoriasis Area and Severity Index [PASI] score of 10.9), joint inflammation (mean SJC of 10, mean TJC of 20), and impaired physical function (mean Health Assessment Questionnaire Disability Index [HAQ-DI] score of 1.3). Overall, 67% of patients had enthesitis. Mean DAPSA (43.8), DAS28 (4.8), GRACE (5.9), PASDAS (6.4), mCPDAI (7.2), patient assessment of pain score (6.3), and C-reactive protein concentrations (1.2 mg/dL) were consistent with highly active PsA. All patients had received at least one prior TNFi, and 12% of patients had received two. Overall, 55% of patients were receiving concomitant methotrexate.

**Table 2.** Baseline demographics and disease characteristics

Characteristic	Guselkumab 100 mg Q8W (*n *=* *189)	Placebo (*n *=* *96)	Total (*N *=* *285)
Age, years	49 (12)	49 (12)	49 (12)
Sex			
Male	86 (46%)	52 (54%)	138 (48%)
Female	103 (54%)	44 (46%)	147 (52%)
BMI, kg/m^2^	29 (6)	31 (7)^a^	30 (6)^b^
SJC, 0–66	10 (7)	9 (6)	10 (6)
TJC, 0–68	21 (13)	18 (11)	20 (12)
PsA duration, years	8.3 (7.8)	8.7 (7.2)	8.4 (7.6)
PASI, 0–72	11.7 (11.9)^c^	9.2 (9.4)	10.9 (11.2)^b^
BSA, %	17.9 (21.5)	13.4 (17.7)	16.4 (20.4)
Pain, 0–100 mm VAS	6.5 (1.9)	6.0 (1.8)	6.3 (1.9)
HAQ-DI, 0–3	1.3 (0.6)^c^	1.2 (0.6)	1.3 (0.6)^b^
Enthesitis (LEI score ≥1)	126 (67%)	64 (67%)	190 (67%)
LEI score, 1–6	2.9 (1.5)	2.7 (1.5)	2.8 (1.5)
DAPSA	45.5 (19.9)^c^	40.6 (15.8)	43.8 (18.7)^b^
DAS28	4.9 (1.0)^c^	4.6 (0.8)	4.8 (0.9)^b^
GRACE, 0–10	6.0 (1.1)^d^	5.6 (1.0)	5.9 (1.1)^e^
PASDAS, 0–10	6.4 (1.0)^f^	6.2 (0.9)	6.4 (1.0)^g^
mCPDAI, 0–12	7.3 (2.2)^f^	7.1 (2.2)	7.2 (2.2)^g^
CRP, mg/dL	1.2 (2.0)^c^	1.2 (2.5)	1.2 (2.2)^b^
Number of prior TNFi			
1	167 (88%)	85 (89%)	252 (88%)
2	22 (12%)	11 (11%)	33 (12%)
Reason for prior TNFi discontinuation			
Efficacy	159 (84%)	79 (82%)	238 (84%)
Safety	30 (16%)	17 (18%)	47 (16%)
csDMARDs			
MTX ongoing at baseline	105 (56%)	50 (52%)	155 (54%)
Any non-biologic other than MTX ongoing at baseline	69 (37%)	36 (38%)	105 (37%)

Data are mean (SD) or *n* (%).

a *n *=* *95.

b *n *=* *284.

c *n *=* *188.

d *n *=* *187.

e *n *=* *283.

f *n *=* *186.

g *n *=* *282.

BMI: body mass index; BSA: body surface area; CRP: C-reactive protein; csDMARD: conventional synthetic disease-modifying antirheumatic drug; DAPSA: Disease Activity Index for Psoriatic Arthritis; DAS28: Disease Activity Score 28; GRACE: Group for Research and Assessment of Psoriasis and Psoriatic Arthritis (GRAPPA) Composite score; HAQ-DI: Health Assessment Questionnaire Disability Index; LEI: Leeds Enthesitis Index; mCPDAI: modified Composite Psoriatic Disease Activity Index; MTX: methotrexate; PASDAS: Psoriatic Arthritis Disease Activity Score; PASI: Psoriasis Area and Severity Index; PsA: psoriatic arthritis; Q8W: every 8 weeks; SD: standard deviation; SJC: swollen joint count; TJC: tender joint count; TNFi: tumour necrosis factor inhibitor; VAS: visual analogue scale.

### Joint-focused composite measure response rates through Week 48

**Figure 1.**
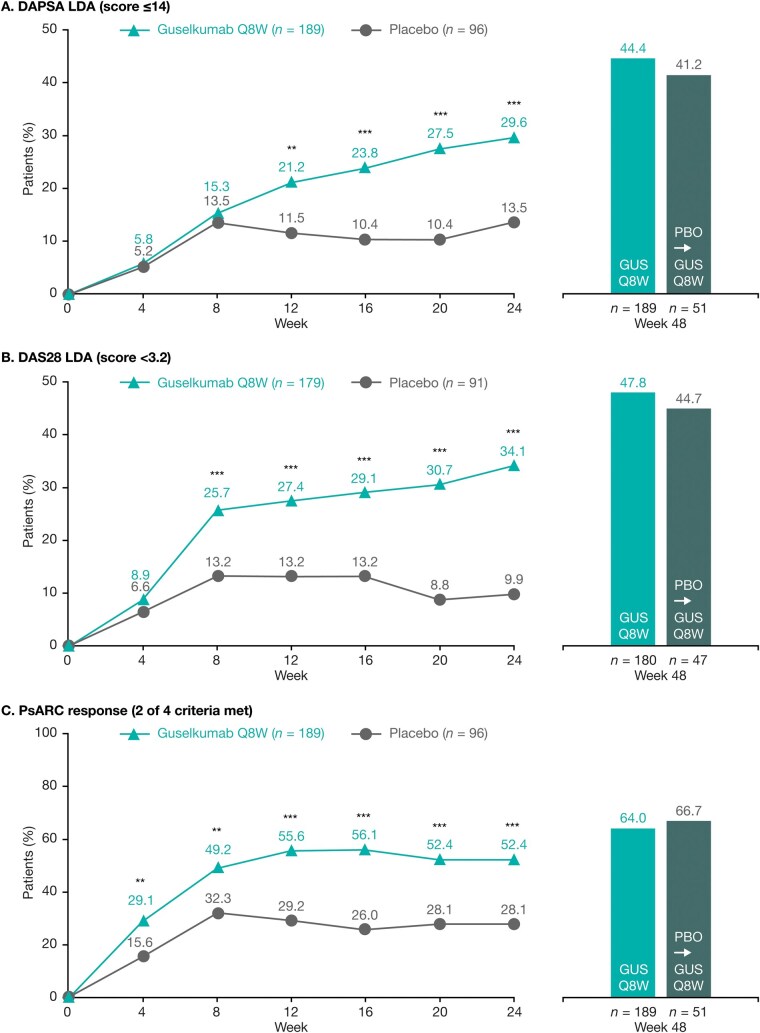
Achievement of (**A**) DAPSA LDA, (**B**) DAS28 LDA and (**C**) PsARC response among patients not fulfilling the outcome criteria at baseline. *P<0.05, **P<0.01, ***P<0.001 *vs* placebo (nominal). Data from NRI analyses. DAPSA: Disease Activity Index for Psoriatic Arthritis; DAS28: Disease Activity Score 28; GUS: guselkumab; LDA: low disease activity; NRI: non-responder imputation; PBO: placebo; PsARC: Psoriatic Arthritis Response Criteria; Q8W: every 8 weeks

A guselkumab treatment effect was observed as early as Week 4 for PsARC, when 29.1% of guselkumab- and 15.6% of placebo-treated patients achieved a PsARC response (Fig. 1). A clear treatment effect was observed at Week 24 across the joint-focused composite measures, with higher proportions of guselkumab- than placebo-treated patients achieving LDA assessed by DAPSA, DAS28 and PsARC response (Fig. 1). Among guselkumab-randomized patients, the proportions achieving each joint-focused composite end point increased after Week 24 through Week 48. At Week 48, 44.4% of patients achieved DAPSA LDA (29.6% at Week 24), 47.8% achieved DAS28 LDA (34.1% at Week 24) and 64.0% achieved a PsARC response (52.4% at Week 24; Fig. 1). For the placebo→guselkumab group, response rates increased substantially after crossover and at Week 48 were similar to those achieved by guselkumab-randomized patients (Fig. 1).

For the more stringent end point, DAPSA remission, a similar trend was observed, with a greater proportion of guselkumab- than placebo-treated patients achieving the outcome at Week 24 (Supplementary Fig. S1). By Week 48, 15.9% of guselkumab-randomized patients achieved DAPSA remission (5.3% at Week 24). In the placebo→guselkumab group, a numerical increase in response rate from Week 24 was observed by Week 48 (Supplementary Fig. S1).

### Multi-domain composite indices response rates through Week 48

**Figure 2.**
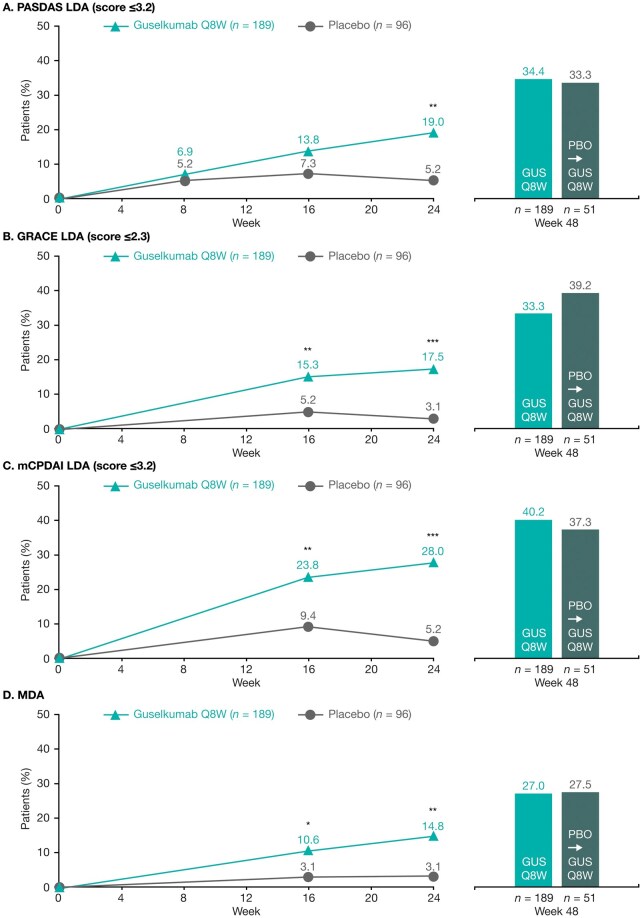
Achievement of (**A**) PASDAS LDA, (**B**) GRACE LDA, (**C**) mCPDAI LDA, and (**D**) MDA among patients not fulfilling the outcome criteria at baseline. *P<0.05, **P<0.01, ***P<0.001 *vs* placebo (nominal). Data from NRI analyses. Time points displayed in the figure panels are determined by the common assessment times of a composite indice’s components and, as such, PASDAS data were only available at Weeks 8, 16, 24 and 48, while GRACE, mCPDAI and MDA data were only available at Weeks 16, 24 and 48. GRACE: Group for Research and Assessment of Psoriasis and Psoriatic Arthritis (GRAPPA) Composite score; GUS: guselkumab; LDA: low disease activity; mCPDAI: modified Composite Psoriatic Disease Activity Index; MDA: minimal disease activity; NRI: non-responder imputation; PASDAS: Psoriatic Arthritis Disease Activity Score; PBO: placebo; Q8W: every 8 weeks

At Week 24, higher proportions of guselkumab- than placebo-treated patients achieved LDA, as assessed by the multi-domain composite indices PASDAS, GRACE, mCPDAI, and MDA (Fig. 2). Among the guselkumab-randomized patients, the proportions achieving each multi-domain composite end point increased after Week 24 such that by Week 48, 34.4% achieved PASDAS LDA (19.0% at Week 24), 33.3% achieved GRACE LDA (17.5% at Week 24), 40.2% achieved mCPDAI LDA (28.0% at Week 24), and 27.0% achieved MDA (14.8% at Week 24; Fig. 2). For the placebo→guselkumab group, response rates for PASDAS LDA, GRACE LDA, mCPDAI LDA, and MDA increased substantially after crossover, and at Week 48 were similar to those achieved by guselkumab-randomized patients (Fig. 2).

Of the multi-domain composite indices that assess remission-related endpoints (PASDAS VLDA and VLDA), greater proportions of guselkumab- than placebo-treated patients achieved the end point at Week 24 (Supplementary Fig. S2). The response rate for these stringent end points continued to increase to Week 48, with 12.7% of guselkumab-randomized patients achieving PASDAS VLDA (4.2% at Week 24) and 11.1% achieving VLDA (3.7% at Week 24; Supplementary Fig. S2). In the placebo→guselkumab crossover group, a numerical increase in response rates from Week 24 was also observed by Week 48 (Supplementary Fig. S2). In line with the joint-focused and multi-domain composite indice findings, higher proportions of guselkumab- than placebo-treated patients achieved low levels of joint or skin disease activity at Week 24 based on the assessment of the individual components of the composite indices (Supplementary Fig. S3).

### Maintenance of response

**Figure 3.**
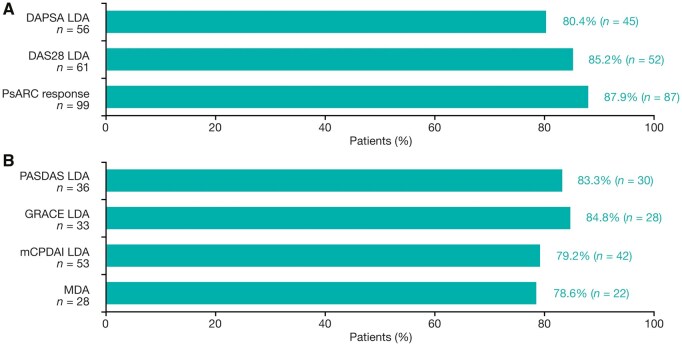
Maintenance of LDA for (**A**) joint-focused and (**B**) multi-domain composite indices at Week 48 among Week 24 responders (guselkumab-randomized patients). A responder was defined as a patient who achieved the given end point. Data from NRI analyses. DAPSA: Disease Activity Index for Psoriatic Arthritis; DAS28: Disease Activity Score 28; GRACE: Group for Research and Assessment of Psoriasis and Psoriatic Arthritis (GRAPPA) Composite score; LDA: low disease activity; mCPDAI: modified Composite Psoriatic Disease Activity Index; MDA: minimal disease activity; NRI: non-responder imputation; PASDAS: Psoriatic Arthritis Disease Activity Score; PsARC: Psoriatic Arthritis Response Criteria

Among guselkumab-randomized patients who achieved LDA at Week 24, as measured by joint-focused composite measures and multi-domain composite indices, most (>80% and >78% of patients, respectively) maintained responses at Week 48 (Fig. 3A and B). Similarly, of the guselkumab-randomized patients who achieved remission-related end points at Week 24, most (70% of patients for DAPSA remission) maintained response at Week 48 (Supplementary Fig. S4). Joint and physical functioning assessments also demonstrated the sustained efficacy of guselkumab at Week 48 among Week 24 responders, with response observed in >72% of patients (Supplementary Fig. S5).

### Predictors of LDA response at Week 24

Of the patient characteristic covariates assessed, score at baseline most often predicted achievement of LDA or remission at Week 24. Specifically, a lower baseline composite index score predicted achievement of LDA for DAPSA (and DAPSA remission), DAS28, PASDAS, GRACE and mCPDAI (Supplementary Table S1). Achievement of DAS28 LDA at Week 24 was also more likely to occur in patients who had received one *vs* two prior TNFi.

## Discussion

Treating patients with TNFi-IR PsA remains a major challenge. Specifically, these patients have decreased ACR20 response rates and lower drug persistence with each successive TNFi [5, 6, 22].

In this *post hoc* analysis of COSMOS, patients with TNFi-IR PsA treated with guselkumab responded better than those treated with placebo across various composite measures of disease activity through Week 24. Though treatment response rates increased through 1 year across all composite indices, the guselkumab treatment effect was observed earlier using joint-focused composite indices than with composite indices comprising multiple domains. Furthermore, response rates were durable, with the majority of guselkumab-randomized patients who achieved responses at Week 24 maintaining response at Week 48. Placebo-randomized patients who crossed over to guselkumab at Week 24 showed improvements in all composite indices, with response rates at Week 48 similar to those observed for guselkumab-randomized patients. Overall, these results align with those observed from a pooled analysis of guselkumab-treated patients in the DISCOVER-1 and DISCOVER-2 trials [17]. Our findings are also consistent with an analysis of the TNFi-experienced patient population in DISCOVER-1 [23]. For example, at Week 24 and among guselkumab-treated TNFi-experienced patients, 14.8% achieved MDA in COSMOS, while 17.1% achieved MDA in DISCOVER-1 [23]. Variations in study designs and data analyses across different studies limit the accuracy of response rate comparisons between different treatments.

Our findings highlight guselkumab as an effective treatment for patients with TNFi-IR PsA regardless of the disease domain affected. This is noteworthy, given that GRAPPA guidelines state that treatment should result in the lowest possible level of disease activity across all affected disease domains [1]. Furthermore, international guidelines for PsA treatment focus on improving quality of life, optimizing functional status, preventing structural damage, and minimizing complications from the disease or its treatment [1, 2, 7]. Therefore, the positive impact of guselkumab across important disease domains, as assessed via composite measures and their individual components, aligns with recommendations for PsA treatment, indicating the relevance of guselkumab in clinical practice.

Composite indices function as a useful tool to comprehensively assess disease activity, by combining patient-reported outcomes and physician-assessed outcomes across multiple domains of PsA. However, some composite indices, such as GRACE and PASDAS, are complex and may be too time-consuming for regular use in clinical practice [24]. For this reason, shortened composite indices with fewer components may be valuable in the real-world setting [24]. In our analysis, composite indices with fewer components appeared to be easier and faster to achieve than comprehensive indices with more domains that are required to improve simultaneously. That is, higher rates of response were observed at earlier time points for assessments such as PsARC (requiring fulfilment of two out of four components) than with composite measures like MDA (requiring fulfilment of five out of seven components), where improvement happens over a longer time period. However, the response rates across composite indices may be affected by not only the number of components comprising each composite index but also the disease domain focus of each composite index. Among the composite indices evaluated with defined disease states or therapeutic thresholds for LDA, those predominantly focusing on the joints, such as PsARC, DAS28 (four components) and DAPSA (five components), resulted in the highest response rates at Week 48 (range, 44.4–64.0%). Meanwhile, multi-domain composite indices, including mCPDAI (six components) and MDA (both including skin and musculoskeletal assessments), GRACE (eight items, including skin) and PASDAS (eight items, with a focus on musculoskeletal domains and with no skin assessment), were associated with lower rates of LDA achievement (range, 27.0–40.2%).

Components of composite indices that are patient-reported may impact the achievement of therapeutic thresholds for LDA [25]. MDA was achieved by only 27.0% of patients at Week 48; while there was a high rate of achievement at Week 48 among the objective component outcomes of MDA, including SJC ≤1 and PASI ≤1, patient-reported outcomes, including pain ≤15, TJC ≤1 and HAQ-DI ≤0.5 response (Supplementary Fig. S3), appeared to limit the achievement of MDA. Patient pain and patient global assessment parameters may also impact the achievement of DAPSA LDA or remission. These findings are consistent with previous studies, which also reported that patient pain, patient global assessment of arthritis activity and TJC limit the achievement of MDA, VLDA or DAPSA remission endpoints [10, 17, 25–27]. Among patients with persistent pain, structural damage to joints established prior to initiation of guselkumab treatment may prevent the achievement of pain ≤15 and impact the patient global assessment of arthritis activity. Consistent with this, the presence of chronic and persistent pain has been reported despite effective control of inflammation with bDMARDs [28, 29]. Furthermore, persistent nociplastic pain resulting from sensitization of the central nervous system may limit the achievement of pain endpoints in PsA [30].

The impact of patient-reported outcomes on achievement of composite end points may be further exacerbated by prior TNFi treatment. In DISCOVER-1, response rates for achievement of the MDA criteria HAQ-DI ≤0.5, patient’s global assessment of arthritis and psoriasis activity ≤20, and pain ≤15 were lower in TNFi-experienced patients than in biologic-naïve patients [31]. In part, this may be due to the immune-modifying effects of TNFi therapy; previous findings from a pooled analysis of the DISCOVER-1, DISCOVER-2 and COSMOS clinical trials highlighted that an IR to TNFi therapies may be associated with dysregulation of the IL-23/T-helper (Th)17 signalling pathway [32]. IL-23 signalling plays a crucial role in driving the production of IL-17A, IL-17F, IL-22, IL-6, and TNFα [33, 34], which contribute to the pathogenic changes in both the musculoskeletal system and the skin of patients with psoriatic disease [35–37]. The pooled analyses demonstrated that baseline levels of IL-22, TNFα and beta defensin-2 (BD-2) were significantly higher in patients with TNFi-IR PsA than in those who were biologic naïve [32]. Dysregulation of the IL-23/Th17 signalling pathway in TNFi-IR PsA patients may therefore account for the lower composite end point response rates typically observed following treatment with guselkumab, when compared with biologic-naïve patients treated with guselkumab [31, 32]. Nevertheless, the results demonstrated that IL-23 inhibition was effective for control of PsA at the immune level, even after IR to TNFi [32].

The presence of comorbid conditions or other baseline factors may also impact the probability of therapeutic end point achievement [11]. In COSMOS, we determined that a worse baseline score for each of DAPSA, DAS28, PASDAS, GRACE and mCPDAI impacted the likelihood of LDA end point achievement for each respective composite index at Week 24 (Supplementary Table S1). The DAPSA baseline score also impacted the achievement of DAPSA remission.

A limitation of these *post hoc* analyses is that they were not powered to detect statistically significant differences between treatment groups. Furthermore, since patients enrolled into COSMOS were required to meet predefined selection criteria based on previous treatment and medical history, the results may not be generalizable to all patients with PsA. Although the number of prior TNF is received and the reason for their discontinuation was documented, data on whether there was a primary or secondary non-response to the TNFi were not recorded.

In conclusion, these *post hoc* analyses of the COSMOS clinical trial provide evidence that patients with TNFi-IR PsA can achieve sustained LDA or remission with guselkumab, as assessed by various composite indices measuring therapeutic response across multiple disease domains. Guselkumab was effective regardless of the focus of the composite indices (joints, skin, enthesitis, dactylitis or patient-reported outcomes). Together, these findings support the role of guselkumab as an important treatment option for the diverse domains of PsA, including in those who have a substantial level of disease activity and an IR to one or two TNFi therapies.

## Supplementary Material

keae586_Supplementary_Data

## Data Availability

The data-sharing policy of Johnson & Johnson Innovative Medicine is available at https://www.janssen.com/clinical-trials/transparency. As noted on this site, requests for access to the study data can be submitted through Yale Open Data Access (YODA) Project site at http://yoda.yale.edu.
