# Supplementary material for: Multi-domain effectiveness of guselkumab evaluated via composite indices through 1 year in patients with PsA and inadequate response to TNFi: post hoc analysis of COSMOS
Source: Rheumatology (Oxford). 2024 Oct 22;64(5):2565–74. doi: 10.1093/rheumatology/keae586 (PMC12048074; doi:10.1093/rheumatology/keae586)
Supplement: keae586_Supplementary_Data [file keae586_supplementary_data.pdf]

Supplementary material

Multi-domain effectiveness of guselkumab evaluated via composite indices through 1 year in patients with PsA and inadequate response to TNFi: *post hoc* analysis of COSMOS

Laure Gossec, Xenofon Baraliakos, Daniel Aletaha, Mohamed Sharaf, Emmanouil Rampakakis, Frédéric Lavie, Clementina López-Medina, Carlo Selmi, Laura C. Coates

Contents

Supplementary table ... 2

Supplementary Table S1. Comparison of therapeutic endpoint achievement at Week 24 between guselkumab and placebo ... 2

Supplementary figures ... 5

Supplementary Figure S1. Achievement of DAPSA remission among patients not fulfilling the outcome criteria at baseline ... 5

Supplementary Figure S2. Achievement of (a) PASDAS VLDA and (b) VLDA among patients not fulfilling the outcome criteria at baseline ... 6

Supplementary Figure S3. Achievement of (a) HAQ-DI ≤0.5, (b) patient pain VAS ≤15, (c) tender entheseal count ≤1, (d) SJC ≤1, (e) TJC ≤1 and (f) PASI ≤1 among patients not fulfilling the outcome at baseline ... 7

Supplementary Figure S4. Maintenance of remission-related endpoints for joint-focused, and multi-domain composite indices at Week 48 among Week 24 responders (guselkumab-randomized patients) ... 8

Supplementary Figure S5. Maintenance of physical functioning, joint or skin response at Week 48 among Week 24 responders (guselkumab-randomized patients) ... 8

Supplementary table

Supplementary Table S1. Comparison of therapeutic endpoint achievement at Week 24 between guselkumab and placebo

**Parameter**	**Covariate**	**Odds ratio**	**95% Wald CI**	***P*-value**
		**LDA endpoints**		
Joint-focused composite measures				
DAPSA LDA	Baseline score	0.936	0.911–0.960	<0.001
	csDMARDs (yes *vs* no)	1.487	0.783–2.824	0.225
	Number of prior TNFi (1 *vs* 2)	3.402	0.948–12.207	0.060
	Guselkumab *vs* placebo	3.791	1.859–7.733	<0.001
DAS28 LDA	Baseline score	0.391	0.257–0.596	<0.001
	csDMARDs (yes *vs* no)	1.838	0.964–3.504	0.065
	Number of prior TNFi (1 *vs* 2)	3.245	1.037–10.156	0.043
	Guselkumab *vs* placebo	6.913	3.114–15.348	<0.001
PsARC response	Baseline PASDAS score	1.022	0.797–1.311	0.862
	csDMARDs (yes *vs* no)	1.488	0.893–2.477	0.127
	Number of prior TNFi (1 *vs* 2)	2.098	0.932–4.719	0.073
	Guselkumab *vs* placebo	2.888	1.687–4.945	<0.001
Multi-domain composite indices				
PASDAS LDA	Baseline score	0.566	0.393–0.818	0.002
	csDMARDs (yes *vs* no)	1.181	0.571–2.442	0.653
	Number of prior TNFi (1 *vs* 2)	1.494	0.417–5.350	0.537
	Guselkumab *vs* placebo	5.023	1.871–13.483	0.001
GRACE LDA	Baseline score	0.641	0.455–0.902	0.011
	csDMARDs (yes *vs* no)	1.481	0.672–3.262	0.330
	Number of prior TNFi (1 *vs* 2)	4.760	0.614–36.873	0.135
	Guselkumab *vs* placebo	7.949	2.327–27.154	<0.001
mCPDAI LDA	Baseline score	0.847	0.730–0.983	0.028

Parameter	Covariate	Odds ratio	95% Wald CI	*P*-value
	csDMARDs (yes *vs* no)	1.665	0.855–3.241	0.134
	Number of prior TNFi (1 *vs* 2)	2.185	0.701–6.811	0.178
	Guselkumab *vs* placebo	7.699	2.937–20.184	<0.001
MDA	Baseline PASDAS score	0.651	0.440–0.964	0.032
	csDMARDs (yes *vs* no)	1.772	0.764–4.106	0.182
	Number of prior TNFi (1 *vs* 2)	8.765	0.514–149.336	0.134
	Guselkumab *vs* placebo	5.323	1.709–16.583	0.004
**Remission-related endpoints**				
Joint-focused composite measures				
DAPSA remission	Baseline score	0.943	0.897–0.992	0.022
	csDMARDs (yes *vs* no)	2.079	0.599–7.219	0.249
	Number of prior TNFi (1 *vs* 2)	3.022	0.186–49.047	0.437
	Guselkumab *vs* placebo	2.649	0.678–10.357	0.161
Multi-domain composite indices				
PASDAS VLDA	Baseline score	0.643	0.335–1.234	0.184
	csDMARDs (yes *vs* no)	1.518	0.375–6.145	0.558
	Number of prior TNFi (1 *vs* 2)	2.055	0.127–33.128	0.612
	Guselkumab *vs* placebo	9.802	0.676–142.152	0.094
VLDA	Baseline PASDAS score	0.471	0.227–0.978	0.043
	csDMARDs (yes *vs* no)	1.271	0.304–5.322	0.742
	Number of prior TNFi (1 *vs* 2)	1.572	0.096–25.708	0.751
	Guselkumab *vs* placebo	8.967	0.626–128.351	0.106
**Components of composite indices**				
HAQ-DI ≤0.5	Baseline score	0.280	0.114–0.691	0.006
	csDMARDs (yes *vs* no)	1.923	0.833–4.438	0.125
	Number of prior TNFi (1 *vs* 2)	5.300	0.672–41.787	0.113
	Guselkumab *vs* placebo	3.311	1.285–8.536	0.013

Parameter	Covariate	Odds ratio	95% Wald CI	*P*-value
Pain ≤15	Baseline score	0.963	0.940–0.986	0.002
	csDMARDs (yes *vs* no)	3.914	1.327–11.549	0.013
	Number of prior TNFi (1 *vs* 2)	7.328	0.401–133.812	0.179
	Guselkumab *vs* placebo	4.394	1.356–14.242	0.014
Tender entheseal count ≤1	Baseline score	0.782	0.590–1.035	0.085
	csDMARDs (yes *vs* no)	2.151	1.048–4.417	0.037
	Number of prior TNFi (1 *vs* 2)	0.648	0.219–1.916	0.433
	Guselkumab *vs* placebo	2.685	1.253–5.754	0.011
SJC ≤1	Baseline score	0.855	0.799–0.915	<0.001
	csDMARDs (yes *vs* no)	1.249	0.712–2.192	0.437
	Number of prior TNFi (1 *vs* 2)	1.837	0.732–4.614	0.195
	Guselkumab *vs* placebo	3.257	1.767–6.004	<0.001
TJC ≤1	Baseline score	0.921	0.874–0.969	0.002
	csDMARDs (yes *vs* no)	1.247	0.557–2.789	0.592
	Number of prior TNFi (1 *vs* 2)	1.229	0.339–4.456	0.753
	Guselkumab *vs* placebo	2.834	1.104–7.276	0.030
PASI ≤1	Baseline score	0.985	0.960–1.011	0.251
	csDMARDs (yes *vs* no)	1.547	0.843–2.839	0.159
	Number of prior TNFi (1 *vs* 2)	0.878	0.346–2.232	0.785
	Guselkumab *vs* placebo	10.106	4.353–23.463	<0.001

Odds ratios and *P*-values generated from a logistic regression analysis.

Baseline PASDAS score was used for adjustment of baseline disease activity for MDA, VLDA and PsARC response (which do not have their own baseline scores).

CI: confidence interval; csDMARD: conventional systemic disease-modifying antirheumatic drug; DAPSA: Disease Activity Index for Psoriatic Arthritis; DAS28: Disease Activity Score 28; GRACE: Group for Research and Assessment of Psoriasis and Psoriatic Arthritis (GRAPPA) Composite score; HAQ-DI: Health Assessment Questionnaire Disability Index; LDA: low disease activity; mCPDAI: modified Composite Psoriatic Disease Activity Index; MDA: minimal disease activity; PASDAS: Psoriatic Arthritis Disease Activity Score; PASI: Psoriasis Area and Severity Index; PsARC: Psoriatic Arthritis Response Criteria; SJC: swollen joint count; TJC: tender joint count; TNFi: tumour necrosis factor inhibitors; VLDA: very low disease activity.

Supplementary figures

Supplementary Figure S1. Achievement of DAPSA remission among patients not fulfilling the outcome criteria at baseline

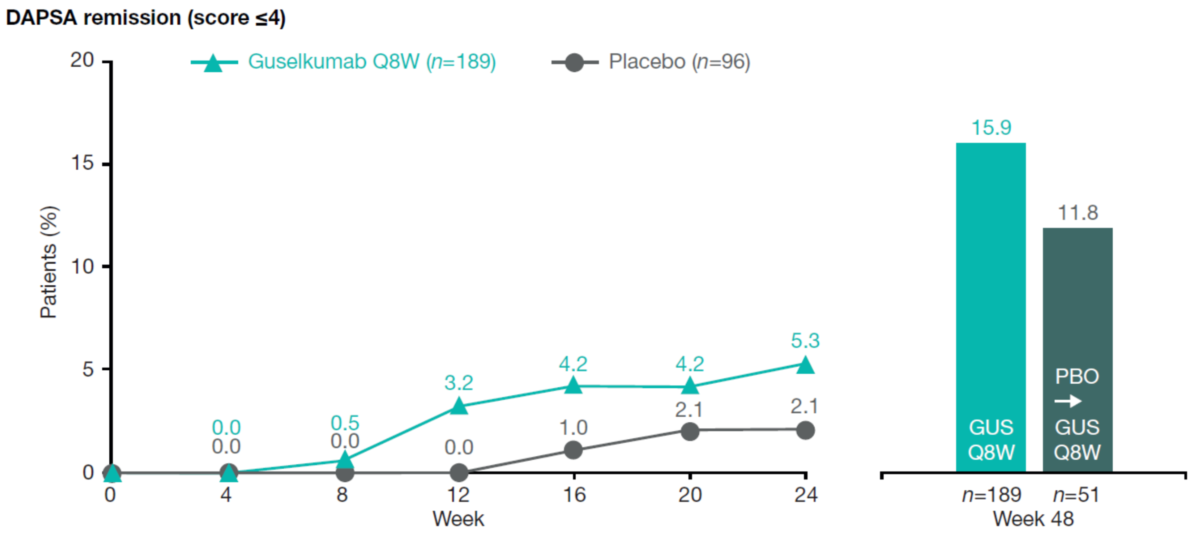

Data from NRI analyses.

DAPSA: Disease Activity Index for Psoriatic Arthritis; GUS, guselkumab; NRI: non-responder imputation; PBO: placebo; Q8W: every 8 weeks.

Supplementary Figure S2. Achievement of (**A**) PASDAS VLDA and (**B**) VLDA among patients not fulfilling the outcome criteria at baseline

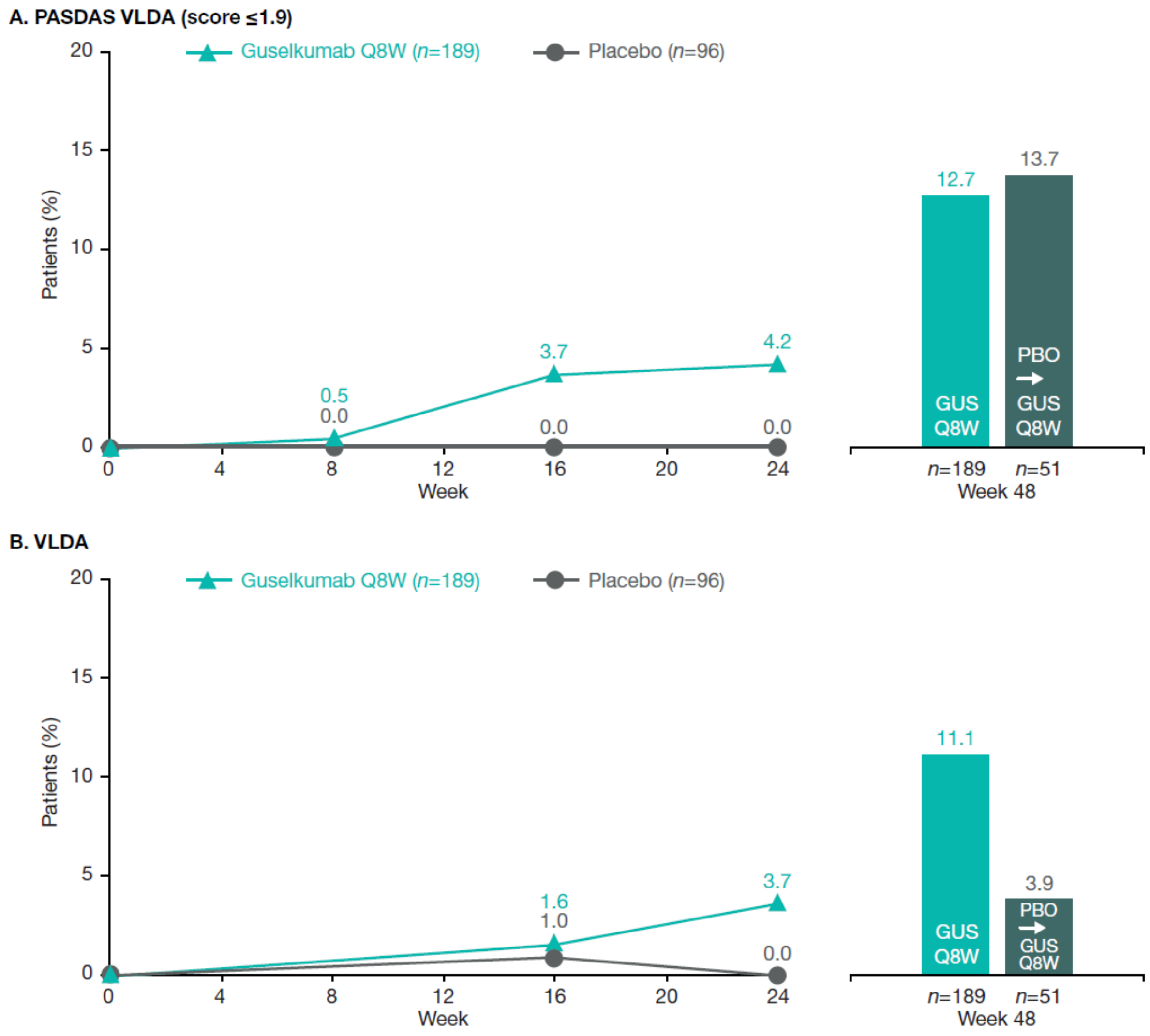

Data from NRI analyses.

GUS: guselkumab; NRI: non-responder imputation; PASDAS: Psoriatic Arthritis Disease Activity Score; PBO: placebo; Q8W: every 8 weeks; VLDA: very low disease activity.

Supplementary Figure S3. Achievement of (**A**) HAQ-DI ≤0.5, (**B**) patient pain VAS ≤15, (**C**) tender entheseal count ≤1, (**D**) SJC ≤1, (**E**) TJC ≤1 and (**F**) PASI ≤1 among patients not fulfilling the outcome at baseline

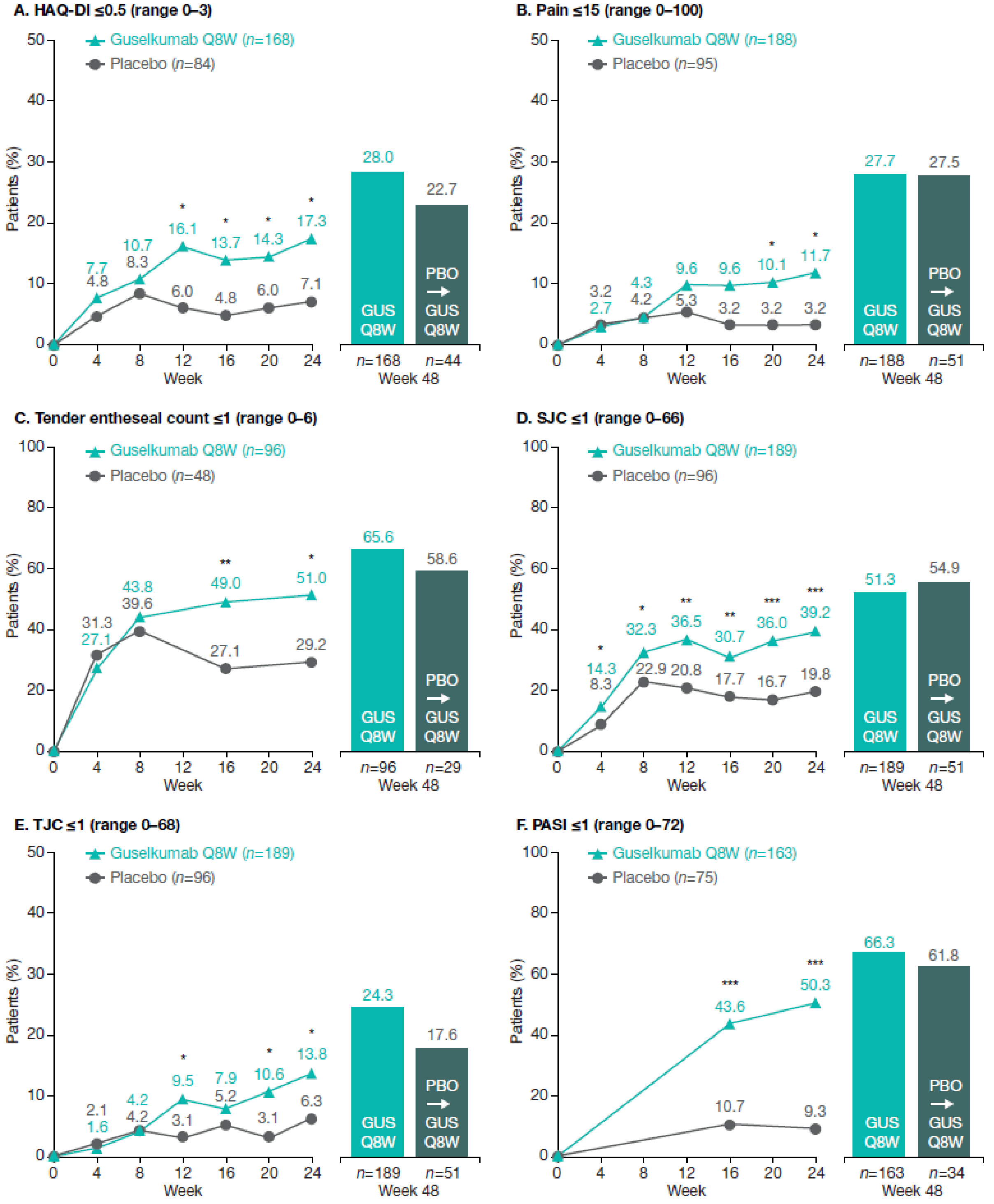

**P*<0.05, ***P*<0.01, ****P*<0.001 *vs* placebo (nominal). Data from NRI analyses.

GUS: guselkumab; HAQ-DI: Health Assessment Questionnaire Disability Index; NRI: non-responder imputation; PASI: Psoriasis Area and Severity Index; PBO: placebo; Q8W: every 8 weeks; SJC: swollen joint count; TJC: tender joint count; VAS: Visual Analogue Scale.

Supplementary Figure S4. Maintenance of remission-related endpoints for joint-focused and multi-domain composite indices at Week 48 among Week 24 responders (guselkumab-randomized patients)

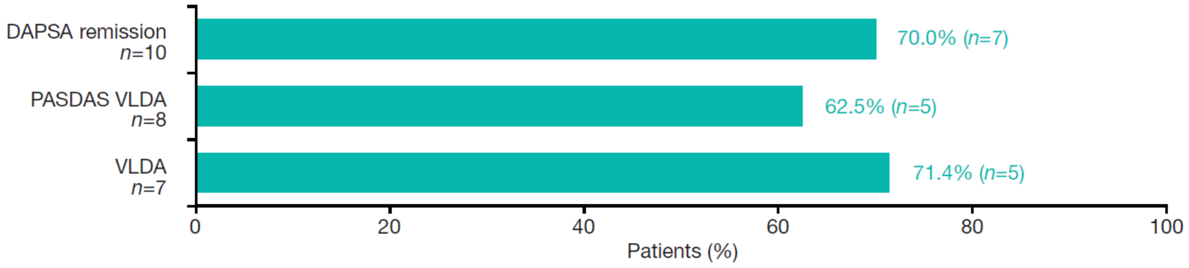

A responder was defined as a patient who achieved the given endpoint. Data from NRI analyses.

DAPSA: Disease Activity Index for Psoriatic Arthritis; NRI: non-responder imputation; PASDAS: Psoriatic Arthritis Disease Activity Score; VLDA: very low disease activity.

Supplementary Figure S5. Maintenance of physical functioning, joint or skin response at Week 48 among Week 24 responders (guselkumab-randomized patients)

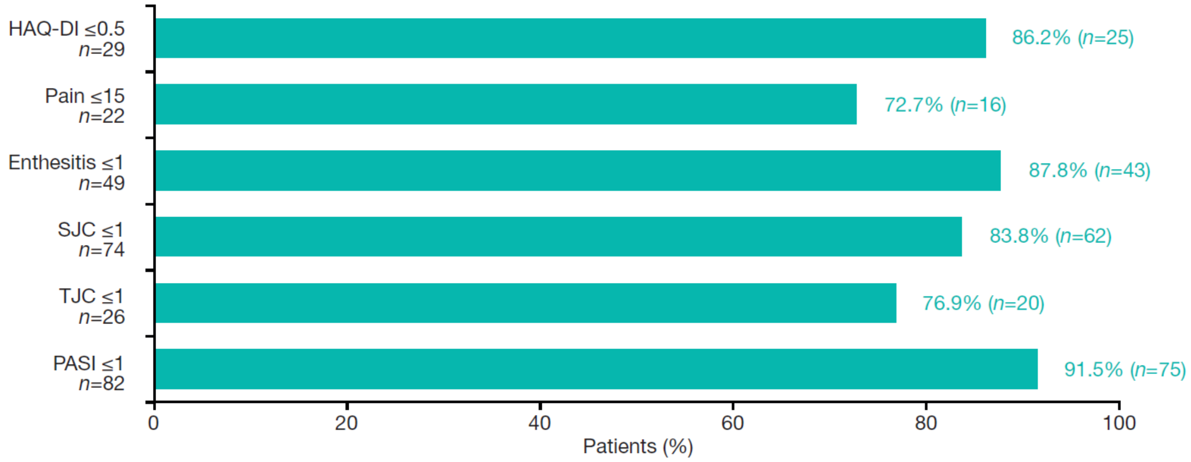

A responder was defined as a patient who achieved the given endpoint. Data from NRI analyses.

HAQ-DI: HAQ-Disability Index; NRI: non-responder imputation; PASI: Psoriasis Area and Severity Index; SJC: swollen joint count; TJC: tender joint count.
